# Bioplastics Toxicity upon Ingestion: A Critical Review of Biotransformation and Gastrointestinal Effects

**DOI:** 10.3390/polym18091091

**Published:** 2026-04-29

**Authors:** Cristiana Fernandes, Helena Oliveira, Teresa Rocha-Santos, Verónica Bastos

**Affiliations:** 1 Centre for Environmental and Marine Studies (CESAM), Department of Chemistry, University of Aveiro, 3810-193 Aveiro, Portugal; cris.fernandes@ua.pt (C.F.); ter.alex@ua.pt (T.R.-S.); 2 Centre for Environmental and Marine Studies (CESAM), Department of Biology, University of Aveiro, 3810-193 Aveiro, Portugal; holiveira@ua.pt

**Keywords:** bioplastic, micro- and nanoplastics, gastrointestinal tract, cytotoxicity, oxidative stress, inflammation, human health

## Abstract

In response to the plastic pollution crisis, bioplastics emerged as a sustainable alternative. However, low degradation rate and abiotic decomposition generate micro- and nanoplastics. These particles enter the food chain, establishing oral intake as a key route of human exposure. This review gathered studies on the biotransformation of bioplastics in the gastrointestinal tract and on their toxicity in human cells and murine models. Most studies focused on polylactic acid particles due to widespread use in food packaging. Under simulated gastrointestinal conditions in vitro, particles were modulated, resulting in cavity and pore formation, fragmentation, lipase competition, protein corona formation, and alterations in the gut microbiota (including *Selenomonadaceae*, *Bifidobacterium*, and *Prevotellaceae*). Also, particle breakdown increases surface area, enhancing interactions with biomolecules and causing higher in vitro and in vivo toxicity. Indeed, pro-inflammatory cytokine secretion, oxidative stress induction, and redox imbalance were found in both models. In mice, alterations in gut microbiota involving *Bacillales* indirectly mediated hepatotoxicity, leading to uric acid and triglyceride accumulation. Furthermore, microbiota adaptation over time was suggested with an increase in microorganisms and the potential conversion of L-lactic into harmful D-lactic acid. Despite limited studies, this review highlighted that ingested bioplastic-derived micro- and nanoplastics can lead to toxic effects.

## 1. Introduction

Plastics production presents growing problems, with recycling rates of less than 10% per year [1,2], considering that production exceeded 430 million tons in 2024. In addition, 90% of plastics are derived from fossil fuels [3] and are therefore associated with increased greenhouse gas emissions and particulate matter [4,5].

In this context, bioplastics have emerged as an environmentally friendly solution [6,7,8,9,10,11] that has been attracting consumers worldwide [12,13,14]. However, current bioplastic production remains very low, at around one per cent of total plastic production [15]. Bioplastics can be classified as bio-based, biodegradable, or both [16]. Bio-based plastics are derived from renewable sources [17,18], while biodegradable plastics degrade into methane, water, and carbon dioxide under the action of microbial consortia [17,19]. Worldwide, the leading manufacturers of bioplastics were China (35.5%), while the European market was led by Germany (around 45%) in 2024 [3]. Increased bioplastics production has been observed; thus, forecasts predict that their manufacturing (2.31 million tons in 2025) may double by 2030 [20]. Bioplastics are normally produced by microbial fermentation, chemical synthesis and enzymatic polymerisation [7,21,22]. As an illustrative example, polylactic acid can undergo microbial fermentation from corn starch, followed by polymerisation. They can then be shaped by extrusion or moulded (by injection or blow moulding) for a wide array of different products [21].

Bioplastics are increasingly being explored for applications similar to those of standard plastics, including in the medical equipment industry, coatings, mulching, automobile manufacturing, and food packaging [16]. Due to the increased awareness of the negative impact of conventional packaging, about 60% of bioplastic output is allocated to the food packaging market [17,18], leading to the phasing out of several conventional plastic-based products within their production lines [23]. Polylactic acid has been widely chosen for this industry, especially for fruit and vegetable packaging and beverage cups, mostly due to its optical clarity [16,24]. Other solutions, such as polyhydroxyalkanoates and polysaccharides, are already available and continue to be improved [19,25].

Even though the fact that biodegradability tends to be perceived as an advantageous feature [26], insufficient knowledge about the correct disposal of such plastics results in increased environmental litter [27,28]. Literature also reported delayed biodegradation rates of some biodegradable plastics in the natural environment [29,30,31]. Abiotic processes, including wave action, photo-oxidation, and abrasion, bring additional concerns to the use of bioplastics [32]. (Bio)degradation may also release additives and smaller particles of various sizes and shapes [33,34]. Smaller fragments include microplastics, defined to be between 1 and 5000 μm in size, and nanoplastics, classified as smaller than 1 μm [35] (still a subject of debate in scientific literature [36]).

Micro- and nanoplastics can spread and accumulate across various environmental niches, posing threats to the environment [37,38,39]. Rivers, surface water, wastewater, biosolids, and organisms, such as invertebrates, are some examples of these matrices [40]. Looking broadly, the adverse outcomes may outweigh the benefits of this eco-friendly solution [41]. Recently, a method coupling pressurised liquid extraction and pyrolysis-gas chromatography-mass spectrometry in combination with thermochemolysis was validated to quantify multiple micro-bioplastics in environmental samples. In this context, samples from municipal wastewater treatment plants in Queensland (Australia) presented concentrations of polylactic acid of up to 0.18 mg g^−1^ in biosolids, 0.15 mg L^−1^ in wastewater influent, and 0.10 mg g^−1^ in sea sediment. Concentrations of polybutylene adipate terephthalate up to 0.10 mg g^−1^ in biosolids, and 0.07 mg L^−1^ in wastewater influent were also measured [42]. Environmentally relevant concentrations of polylactic acid, polybutylene adipate terephthalate, polyhydroxyalkanoates, and polybutylene succinate were reported, varying between 0.054 and 180 μg L^−1^ in wastewater, and in marine and reservoir environments [43].

To date, the literature has paid limited attention to the presence and fate of micro- and nanoplastics derived from bioplastics in the human organism, in contrast to conventional plastics [35,44,45]. However, in light of the growing interest in bioplastics as an emerging alternative, a growing body of evidence is now addressing this critical gap (e.g., [46,47,48,49]). To illustrate, a post-mortem study revealed the accumulation of fibre polylactic acid in human thyroid and brain cavity samples, suggesting the potential to cross the blood–brain barrier and be distributed throughout the circulatory system [46]. The presence of polylactic acid was also detected in placenta, meconium, faeces, and breast milk [47], and polycaprolactone was detected in the placenta and meconium samples [48]. Finally, a study highlighted the need to reduce exposure to microplastics in men of reproductive age, reporting the presence of polylactic acid in 2.2% of the semen samples analysed [49]. Multiple routes of chronic exposure to conventional micro- and nanoplastics in humans include dermal contact, inhalation, ingestion [50,51,52], and potentially through pregnancy [47,48], which are also considered for micro- and nanoplastics derived from bioplastics [40]. Evidence indicates ingestion as the prevailing route [53] and advises against consuming up to five grams of microplastics per week, raising concerns about their effects [54,55,56]. These fragments can accumulate in the food chain [39,40], potentially allowing micro- and nanoplastics derived from bioplastics to biomagnify, reflecting concerns similar to those of conventional plastics [57,58,59].

Research carried out to understand the digestibility and impact of bioplastics in the human digestive system adopted approaches based on in vitro digestion models that mimic relevant gastrointestinal conditions, in vitro cell models, and in vivo experiments, for instance, involving rodents (e.g., [60,61,62]) [35]. At present, the mechanisms of human digestibility of bioplastics remain poorly understood. Biopolymer-dependent exposure of micro- and nanoplastics in the gastrointestinal tract drives interactions among lipids, biomolecules, and the microbiota, which may modulate the morphology and surface area of these materials. This is reflected in surface modification, the absorption and aggregation of biomolecules, hydrolysis, and oligomerisation, which ultimately results in the formation of smaller nanoplastics and affects bioavailability [62,63,64]. Cytotoxicity, intestinal epithelial lesions, altered mucus production, impaired permeability, inflammatory responses, and dysbiosis were among the outcomes observed following bioplastic treatment [65,66,67]. Although mainly linked to conventional micro- and nanoplastics, prolonged exposure seems to be a contributing factor to increased rates of gastritis, gastritis-related cancers, inflammatory bowel disease, or even colorectal cancer [68]. As such, the digestive system stands out as the key target for research on ingested bioplastic fragments [65,66].

Given their emergence as an environmentally friendly alternative, there is a need to understand the potential impacts of bioplastics on human digestion, especially since available toxicological data suggest that bioplastics may be less safe or exhibit more pronounced effects than conventional plastics (e.g., [69]). Knowledge of how bioplastics transform and affect digestibility is limited, and there are few studies using advanced models to evaluate harmful effects, given the heterogeneous bioplastic materials, conditions, doses, and models employed.

Accordingly, the major purpose of this review was to provide a critical analysis of the transformation of bioplastics in the gastrointestinal tract, including cellular toxicity, compromised barrier function, and associated outcomes. Hence, we outlined specific goals: (i) provide insight into the gastrointestinal digestion of bioplastics in relevant models of gastrointestinal conditions; (ii) review the potential cytotoxicity of bioplastics in pertinent in vitro models and alterations in the barrier (such as permeability and integrity), and related endpoints/mechanisms, which could be translatable to humans in the digestive system; and (iii) summarise the biodistribution behaviour and toxicity of ingested micro- and nanoplastics derived from bioplastics in in vivo studies in the digestive system, thereby providing a basis for future risk assessment efforts.

## 2. Methods

Bibliographic research on relevant studies was conducted using Google Scholar and Scopus. Additionally, a manual search was conducted using cross-reference analysis to incorporate studies that were not captured in the preliminary selection. The following keywords relevant to the three topics covered in this review were: “bioplastics”, “bio-MNPs”, “biobased”, “biodegradable”, and specific polymers. Combined terms included: “in vitro”, “gastrointestinal”, “simulated gastrointestinal”, “digestive system”, cytotoxicity”, “MTT”, “resazurin”, “TEER”, “LDH”, “cell viability”, “metabolic activity”, “barrier integrity”, “barrier permeability”, “in vivo”, mice”, “mouse”, “rat”, toxicity”, “inflammation”, and “human health”.

A selection of articles written in English that fit the purpose of this review was considered, while excluding those that addressed biopolymers as either nanocarriers or devices for medical purposes. Refined results included studies of bioplastics subjected only to simulated gastrointestinal conditions, plus in vitro toxicity testing on system-relevant cells and in vivo toxicity testing in rodent models of the digestive system. As a result, 25 studies (including nine with cross-references) met the inclusion criteria based on title, abstract, and then full-text reading.

## 3. State-of-the-Art of the Digestion of Bioplastics in Humans in Simulated Gastrointestinal Tract Conditions

**Figure 1:**
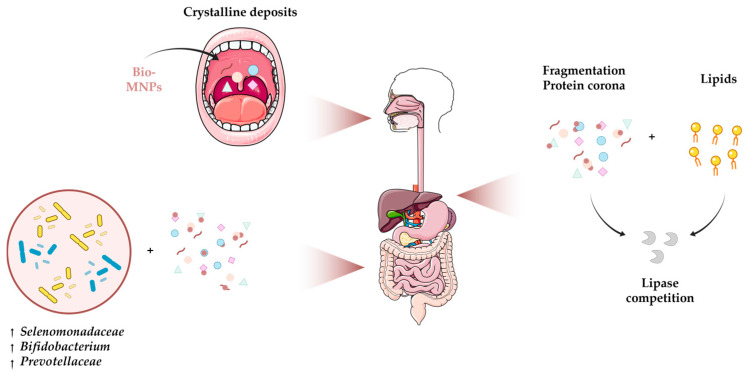
Biotransformation of micro- and nanoplastics derived from bioplastics (bio-MNPs) upon ingestion. Adapted from Servier Medical Art (https://smart.servier.com, accessed on 25 February 2026), licensed under CC BY 4.0 (https://creativecommons.org/licenses/by/4.0/), and NIAID NIH BioArt Source (https://bioart.niaid.nih.gov/bioart/349, accessed on 5 March 2026). ↑ Increased.

The growing field of bioplastics as more sustainable alternatives, together with evidence of human biomagnification [57], raises questions about their biotransformation, fate, and outcomes when ingested by humans [70]. Owing to its extensive use in food contact products, polylactic acid is among the most studied biopolymers [71]. To simulate human digestion of particles, leachates, or migration extracts ingested orally, relevant in vitro fluids (such as salivary, gastric, and intestinal) and colonic fermentation have been employed in the laboratory [60,62,63,69,72,73,74]. These fluids are known to modulate the physical and chemical properties and to influence the systemic absorption of conventional plastics [75,76]. An overview of the biotransformation of micro- and nanoplastics derived from bioplastics is shown in Figure 1.

Simulated oral exposure triggered early crystalline-deposit formation in polylactic acid polymer, without other expected signs of biodegradation, as depicted in Figure 1. Subsequently, Raman spectroscopy and field emission scanning electron microscopy revealed cavities and pores developed after treatment with gastric fluid and colonic fermentation. Modifications in the polymer structure observed after exposure to gastric fluid were correlated with the acidic environment [60]. In addition, the roughness of the surface observed by scanning electron microscopy could promote more molecular interactions with biomolecules [62]. Overall, evidence suggests that the transformation rates of bioplastics during gastrointestinal transit vary across bioplastics depending on the compartment and employed conditions. For instance, a comparative study demonstrated that polylactic acid degradation began in the small intestine rather than the stomach, likely because acidic conditions could result in slower degradation rates than alkaline conditions [72], as opposed to the previous hypothesis [60]. After exposure to colonic fermentation, both polylactic acid and polycaprolactone were decomposed by the gut microbiota, yielding oligomers (Figure 1), possibly as a result of ester bond cleavage [72]. Scanning electron microscopy revealed breakdown into smaller particles in poly(butylene succinate-co-glycolate) and polylactic acid after exposure to simulated gastrointestinal fluids [74]. Evidence suggests these fragments may enter the circulatory system and bioaccumulate in other tissues. Consequently, they can affect other organ systems throughout the organism’s body, and exhibit increased toxicity [62] (as discussed in Section 5).

Similar to standard plastics [77], the interactions of bioplastics with lipids were also analysed, due to their potential impact on lipid digestion [78]. According to a molecular dynamics study, polylactic acid adhered to dipalmitoyl phosphatidylcholine bilayers, achieving high theoretical values for molecular interaction. Thus, absorption resulted in increased surface roughness and a reduction in the thickness of the lipid bilayer [79]. The modified surfaces, therefore, have the potential to bind lipids or to modulate lipid droplet structure. It can limit the area accessible to lipases in a time-dependent process [62,63]. To illustrate, poly(lactic-co-glycolic acid) showed that sustained interaction with lipid emulsion could impair lipid digestion over time, as evidenced in a simulated gastric fluid, resulting in lower levels of free fatty acids [63]. The competitive binding and accessibility of lipase by bioplastics in the stomach were also demonstrated in a different study, in which polylactic acid hydrolysis by lipase promoted competition for the enzyme (Figure 1). Their interaction was correlated with the oligomer weight loss during digestion and the steady formation of self-assembling nanoplastics, which can disperse in the lumen [62].

Few studies emphasised degradation and co-ingestion interactions involved in bioplastic digestion. Further research is required in conditions that better simulate bioplastic degradation or leaching to underly the modifications, mechanisms, and potential effects of bioplastics on human health after ingestion [73]. Indeed, we are primarily confronted with micro- and nanoplastics in their degraded form, rather than in their pristine state [80]. A simulation model of photodegradation of polylactic acid/polybutylene adipate terephthalate films identified structural shifts, such as particle fragmentation and oxidation products, ascribed to the release of free radicals, prior to exposure to gastrointestinal fluids. Hence, ageing can enhance the susceptibility of bioplastics to digestive degradation, since it increases the contact area and the likelihood of interaction with biomolecules. Indeed, those effects were intensified after gastrointestinal exposure, evidenced by changes in colour, hydrophilicity, weight loss, increased porosity, and particle formation smaller than 1 µm [69].

In the meantime, there is a gap in the literature on food matrices combined with bioplastics, a key scenario in daily life [73]. Migration studies have revealed that particles and additives can migrate and leach, often to a greater extent than in conventional plastics. Such extracts have been used in toxicological studies, which have shown adverse effects on human health (e.g., [81,82,83,84]). In this vein, a recent study assessed the interaction of polylactic acid and a petroleum-based plastic (polyethylene terephthalate) with seafood proteins to evaluate bioaccessibility. The findings showed no statistically significant differences, suggesting minimal interaction in this specific context. However, the potential effects cannot be ruled out without further investigation [73]. Also, within the scope of bioavailability/bioaccessibility, a risk assessment was carried out for 12 metals from bioplastics, namely polylactic acid, polyhydroxybutyrate, and poly(3-hydroxybutyrate-co-3-hydroxyvalerate). Raw, marine-aged, and artificially metal-loaded laboratory matrices were exposed to gastrointestinal conditions using a dialysis membrane, after which metal content was quantified using high-resolution inductively coupled plasma mass spectrometry. Overall, raw microplastics showed no bioaccessible or bioavailable content and, as a result, did not pose a risk of ingestion. Ageing material showed bioaccessible iron and manganese, but only manganese was bioavailable, indicating its potential for ingestion. Furthermore, the bioavailability of cadmium, chromium, cobalt, and antimony in artificially loaded metal matrices was observed. However, a potential carcinogenic risk was identified only for chromium [44].

The accumulation of organic and microbial matter and the formation of biofilms coating particle surface during digestion have been observed, supporting the idea of the formation of a protein corona (see Figure 1). This evidence was supported, by way of example, through an increase in the weight of the particles at some stages of digestion [60,63]. Currently available results remain limited and heterogeneous; however, this phenomenon was previously reported for polylactic acid, polycaprolactone, and polybutylene adipate terephthalate films (e.g., [60,72]). It is therefore known that this protein layer can reshape the signature of the biopolymer [85], strongly influencing interactions with biomolecules and gut microbiota, ultimately resulting in aggregation and recognition by the immune system [86]. Studies have described fluctuations in alpha diversity and microbial composition [60,72]. Variations in bacterial genera, such as *Selenomonadaceae* [72] and *Bifidobacterium* [60], and in families, such as *Prevotellaceae* [72] (Figure 1), were identified through analysis of the 16S ribosomal RNA gene. Another study pointed to subtle modulation, without signs of health impacts [60]. Altogether, it appears that microbiota colonisation contributes to microbial breakdown, reducing particle size, while promoting oligomeric by-products, likely due to the presence of microbial carbohydrate esterases that can catalyse ester bond cleavage [60,72].

The above-mentioned modulations may disrupt normal cell function, potentially leading to adverse toxic effects and compromised barrier integrity, as addressed in Section 4. While a recent review (Malafeev, 2025) addressed the cytotoxicity of bioplastics across various physiological systems [35], the present work provides a dedicated and in-depth focus on the gastrointestinal tract. This review supplements the existing literature with a more granular analysis of the gastric environment, incorporating the most recent studies that are critical to the current state-of-the-art.

## 4. Cytotoxic Effects, Cellular Responses, and Intestinal Barrier Integrity Triggered by Bioplastic Exposure in Key Gastrointestinal Cell Models

Micro- and nanoplastics derived from bioplastics, along with the release of additives into the gastrointestinal tract, may result in their uptake by the intestinal mucosa or epithelium [87]. Proposed routes for micro- and nanoplastics uptake include endocytosis, microfold cell-mediated transcytosis, pinocytosis, or paracellular transport [73]. Site-specific locations were reported in the patches of Peyer, which are abundant in immune system cells known as microfold cells [57]. Literature also pointed to the possibility of internalisation by other immune cells [69], and their ability to cross the bloodstream and target distal organs [62]. Hence, although sparse, research on the toxic effects of bioplastics ingested by humans has recently been expanding in parallel with the growing efforts to elucidate the underlying mechanisms. An outline of recent studies evaluating cytotoxicity and barrier impact in response to micro- and nanoplastics derived from bioplastics treatment in relevant digestive system cells, both human- and rodent-derived, is presented in Table 1. For this review, attention was placed on the type of bioplastic particles or films, exposure conditions, size, cell models, dose and time exposures, cytotoxic effects, intestinal barrier integrity, permeability, and other associated outcomes, wherever available.

A large group of the studies listed in Table 1 was conducted using polymers exposed to some form of degradation or digestion treatment. Notable cases involved mimicking in-soil migration/degradation [67,91], hot beverages [83,89], gastrointestinal conditions [69,90], and migration in food matrices [84,89], photodegradation [67,69,91], and additive extraction [82]. The mentioned conditions are closely related to a real-life exposure scenario in intestinal, immune or hepatic cells. Overall, polylactic acid was the most studied bioplastics, due to its application in food packaging [71]. Additionally, acute toxicity and the associated underlying mechanical pathways were assessed, extending up to 72 h.

Human translation of biological outcomes has been carried out primarily using two-dimensional monolayer cells to study the interaction and impacts of micro- and nanoplastics derived from bioplastics in the gastrointestinal tract [83,93]. Most two-dimensional models are well established and easy to handle, making them suitable for cytotoxicity assays [94]. In particular, cells derived from human colorectal adenocarcinoma (Caco-2 cells) have been widely used in this vein [67,83,87,88,89,90]. Actually, after cell differentiation, tight junctions, microvilli, enzymes, and enterocyte-like transporters enable the assessment of cell structure and permeability in Transwell systems [95]. Moreover, another study conducted in this framework employed murine epithelial-like intestinal cells (SCT-1) [77]. Human monocytes (THP-1) were also used to assess the cytotoxicity of bioplastics, as they can recognise, engulf and degrade the particles [67,69]. Beyond that, the liver is a plausible target organ given its role in metabolisation and absorption of micro- and nanoplastics [96]. Hence, murine (BNL CL.2 [77]) and human (L-02 or LO2 [67,84], HepG2 [82,87,88], HepG2/C3A [91] and HepaRG [87,88]) hepatocytes, as well as murine hepatic macrophages (lmKC [77]), were likewise employed to assess the hepatotoxicity of micro- and nanoplastics derived from bioplastics. However, the mucous (primary point of contact with particles) and interactions with other cell types are not covered in these two-dimensional models [97,98]. Co-cultures with mucus-producing cells (HT-29 or HT29-MTX [83,87]) or immune cells (Raji-B [87]) were also adopted to mitigate these limitations. Furthermore, co-culture intestine-liver systems, such as Caco-2/HepaRG cells, were adopted to study possible toxic effects of micro- and nanoplastics derived from bioplastics [87]. Higher complexity models, including differentiated induced pluripotent stem cells into epithelial cells, which mimic heterogeneity in human epithelium, were also included in a single study [92].

Since the epithelium is the primary line of defence, the death of epithelial cells can impair cell permeability and barrier function. Under these circumstances, the gut can become prone to diseases [99]. Particle size seems to be a critical factor influencing cell viability, with smaller particles causing higher toxic levels, which also depends on exposure time, cell line, and concentration, as previously reported for conventional micro- and nanoplastics [100]. For example, a study applied photodegradation conditions and simulated gastrointestinal conditions to polylactic acid/polybutylene adipate terephthalate films. Ultrafiltration-collected particles were smaller, resulting in near-total lethality at higher concentrations in THP-1 monocytes, with a maximum effective concentration of 293 mg L^−1^. This contrasted with centrifugation, in which larger particles were collected, and higher concentrations halved viability. These differences were attributed to the increased contact area and the resulting greater surface reactivity. Moreover, it was observed that the particles collected by ultrafiltration exhibited similar or even stronger cytotoxicity compared to traditional plastic-derived particles (such as polystyrene and poly(methyl methacrylate)) [69].

In a study involving polylactic acid particles of different sizes, ranging from 5 × 10^7^ to 2.5 × 10^10^ µm^2^ particles mL^−1^, it was also noted that the smaller particles had greater toxic effects. This was observed in HepG2 and HepaRG hepatocytes and in differentiated Caco-2 cells over time, suggesting higher reactivity. In this case, permeability was not impaired, even though smaller particles were internalised. Findings also suggested that cellular absorption may be promoted by reducing particle size. Further, it could be driven by lipophilic interactions at the cell membrane. This was suggested because polylactic acid particles were distributed across the membrane and were likely to intercalate. Hence, further research should be conducted on the potential adverse effects of nanoplastics on impairing cellular functions and their interactions, considering their potential to target other organs [88].

Environmentally relevant or routine exposure levels were also considered [83,84,89,90]. Based on transmission electron microscopy analysis, polylactic acid particles were released during tea preparation simulation, with an estimated concentration of 1 M polylactic acid per tea bag. A high level of absorption was observed when Caco-2 cells were co-cultured with HT29 cells. Mucus-producing cells internalised 100%, while intestinal cell internalisation was lower, according to confocal microscopy and flow cytometry, and persisted for up to 72 h. A reduction in transepithelial electrical resistance was observed at 3 h, but not at later times, an effect explained by the continuous proliferation and division of HT29 cells, which may have allowed them to repair the initial injury. For this reason, putative effects on the intestinal epithelium in a real-life setting should not be disregarded [83]. In addition, heating of polylactic acid-coated takeaway food containers was simulated, resulting in increased release of micro- and nanoplastics. The raw leachate and supernatant reduced cell viability in Caco-2 cells, whereas resuspended microplastics had the opposite effect. Cytotoxicity was associated with a decrease in membrane potential and with changes in genes involved in metabolism, biogenesis, signal transduction, and intracellular homeostasis, as assessed by transcriptomics [89].

The effects of polybutylene adipate terephthalate-starch blend extracts, obtained by simulating food exposure to acetic acid and ethanol, were studied, as chemicals can migrate from packaging to food products during accommodation or storage. Cytotoxicity effects were observed in L-02 liver cells at medium-higher percentages of migration extracts, except for the 95% (*v*/*v*) ethanol simulation, which reduced viability even at lower percentages. Aspartate aminotransferase and alanine transaminase (liver damage markers) increased with the exposure of migration extracts in liver cells. Inflammation was linked with an increased secretion of tumour necrosis factor-alpha, interleukin-6, and interleukin-1beta cytokines. Moreover, redox imbalance was evidenced by increased oxidative stress, decreased antioxidant enzyme activity, and mitochondrial damage. Thus, the study raises questions about food packaging safety [84]. Evidence from particles varying in size in differentiated monolayers of Caco-2 cells suggested that, after exposure to gastrointestinal conditions, cell viability was not affected. However, undigested particles exhibited higher cellular interaction with the intestinal cells. This appeared to be associated with the agglomeration of particles with organic matter in the intestinal fluid, as observed in transmission electron microscopy imaging, thereby reducing cellular uptake as fewer particles were available for cell absorption. On the other hand, the transport of all particles across the intestinal barrier increased slightly. In future work, the study recommended using particles previously exposed to digestive fluids, recognising that human beings are not typically exposed to pristine particles [90].

Single-use household items were also included to investigate the impact of additives in polylactic acid and polyhydroxybutyrate bioplastics. Extracts were analysed by high-performance liquid chromatography coupled with high-resolution mass spectrometry, detecting an average of 123 and 121 additives in polylactic acid and polyhydroxybutyrate, respectively. However, only 63 compounds were quantified, comprising concentrations of 4–24 µg g^−1^ in polylactic acid items and 10–11 µg g^−1^ in polyhydroxybutyrate items. The most detected additives were plasticisers and chemical intermediates, including phthalates. Cytotoxicity in HepG2 liver cells was not observed; however, treatment with additives increased oxidative stress by elevated reactive oxygen species levels [82].

Microplastics can accumulate in the groundwater, eventually entering the food chain. A comparative study evaluated commercial bioplastics, simulating biodegradation in soil, including polyglycolic acid, polylactic acid, polybutylene succinate, poly(butylene carbonate), polybutylene adipate terephthalate, and aged polyglycolic acid, with particle size up to 800 nm. Among these, polylactic acid displayed the highest environmental mobility, while polyglycolic acid and polybutylene succinate showed the lowest microplastic release, indicating a reduced environmental impact. The degradation products consisted mainly of organic substances. Cytotoxicity was assessed on liver (LO2), intestinal (Caco-2) and monocytic (THP-1) cell lines at concentrations up to 100 mg L^−1^. Both polyglycolic acid and aged polyglycolic acid showed lower biotoxicity, with photoageing contributing to a reduction in polyglycolic acid’s cytotoxicity. Bioplastics did not show meaningful antiproliferative effects in Caco-2 cells, suggesting they can be broken down by gut enzymes in the gastrointestinal tract, generating non-toxic byproducts. It was observed, however, that antiproliferative effects in THP-1 cells (except for aged polyglycolic acid) were presumably due to macrophage phagocytosis, as demonstrated by Nile Red staining, in which polyglycolic acid was engulfed over time [67]. These findings diverge from a previously mentioned study (conducted under different conditions), which suggested that aged polylactic acid/polybutylene adipate terephthalate films might release more toxic micro- and nanoplastics in THP-1 cells under gastrointestinal conditions. Although structurally different, the concentrations used to evaluate cell viability of films were higher (up to 1000 mg L^−1^), which may be higher than those found in the environment [69], compared to concentrations employed for polyglycolic acid (up to 100 mg L^−1^) [67]. Ageing was carried out for 15 days in the case of films [69], against the 3 months of aged polyglycolic acid [67]. However, free radicals and oxidation products were generated within the functional groups, which may have led to a significant decrease in viability upon exposure to films. It should therefore be noted that the gastrointestinal exposure of the films could have contributed to the formation of smaller particles and leachates, potentially increasing their toxicity [69]. An additional study incorporated a combination of additives in polybutylene adipate terephthalate films (carbon black/hindered amine light stabiliser, and carbon black/vitamin E) and simulated degradation in soil followed by photoageing. Findings showed a decrease in viability dependent on the dose in HepG2/C3A cells; however, up to 250 µL mL^−1^, it remained above 80%. At this concentration, genotoxicity was also not observed, indicating that polylactic acid/polybutylene adipate terephthalate films appear safe under these conditions [91].

Relevant Caco-2 co-culture models involving the immune system, mucus-producing cells, and hepatocyte cells were used to evaluate the biological effects of submicrometric and nanometric polylactic acid-based particles. The uptake of particles by the epithelium cells in Caco-2 monolayer, Caco-2/Raji-B, and Caco-2/HT29-MTX models occurred without impairment. In addition, uptake of smaller particles by hepatocytes was higher in the Caco-2/HepaRG co-culture model. Larger particles triggered changes in gene expression related to oxidative stress and inflammation in Caco-2 cells, while smaller particles affected only gene expression related to inflammation. Both deregulated inflammatory genes in HepaRG and showed differences in xenobiotic metabolism genes. Indeed, pro-inflammatory cytokine release was detected, without any dose-dependent pattern [87].

A gut model deriving from induced pluripotent stem cells was used to assess the toxicity of micro- and nanoplastics derived from bioplastics. Polylactic acid at 125 µL mL^−1^ was internalised without compromising barrier integrity, and was found in the cytoplasm near the nucleus. There were also low levels of protein on the surface; however, emphasis was placed on the importance of the protein corona formed during digestion when evaluating cytotoxicity [92]. A study conducted with polylactic acid and cellulose acetate particles across different murine cell lines (lmKC, J774A.1, STC-1, and BNL CL.2) found that up to 100 particles per cell did not induce cell toxicity. A dose-dependent increase in reactive oxygen species levels was observed in macrophages after exposure to polylactic acid, while all cell lines induced reactive oxygen species after exposure to cellulose acetate. The study suggests a possible impact of these modifications on the axis between the liver and the gut [77].

Cytotoxicity, side effects, and underlying mechanisms are elucidated through in vitro studies. Nevertheless, to better encompass the toxic effects of ingested bioplastics in the digestive system and their bioaccumulation, an in vivo approach is required. Hence, the topic and studies carried out in that framework were addressed in Section 5.

## 5. Biodistribution, Modulation, and Toxicity of Ingested Bioplastics in the Digestive System in Murine Models

Studies in in vivo models provide a better understanding of the toxic mechanisms and biodistribution processes involved in the ingestion of micro- and nanoplastics [101,102]. In fact, murine models have been used in toxicity studies [102,103] due to their anatomical, physiological, and genetic similarity to humans [104]. The cellular and molecular effects and the fate of micro- and nanoplastics have been explored in mice and rats [101]. Recent reviews on micro- and nanoplastics intake have highlighted toxic side effects induced by oxidative stress, inflammation, or changes in metabolism, further reinforcing their relevance as biological models [105,106]. A summary of current knowledge on toxic outcomes in the gastrointestinal tract following bioplastic ingestion in murine models is presented in Table 2. Emphasis was placed on the type of biopolymer used, particle size across different gastrointestinal compartments, exposure doses and durations, and adverse effects and relevant outcomes within the digestive system.

As we can observe in Table 2, polylactic acid was the most studied bioplastic to assess toxic outcomes in the gastrointestinal system in murine models after ingestion. Rodents were mainly exposed via oral gavage, except in one case, which involved a dietary strategy. In the first case, dosage monitoring may be more accurate, whereas the second one may be closer to real-life exposure and better reflect interactions with oral cavity surfaces [112]. Given that human exposure is estimated to be between 0.1 and 5 g per week [56], the equivalent ingestion can be converted to a standardised, body weight-based daily dose and further extrapolated to rodent models using a correction factor based on body surface area. Based on this approach, the equivalent dose in mice is estimated to range from 2.93 to 147 mg kg^−1^ day^−1^, as observed in the majority of studies, with higher doses used to assess potential risks. Doses within this range were employed in various studies (e.g., [62,64,65,74,107,108,110]), also considering human yearly exposure, although higher doses for toxic effects were considered. Notably, Bao et al. used this approach and considered a single dose of 200 mg kg^−1^ in mice as a human-equivalent dose [111]. Also, a lower concentration was employed (e.g., [109]). Usually, acute toxicity (response within 7 days) and subacute/subchronic administered doses were considered for evaluation. Meanwhile, another study provided assessments over a longer period (up to 3 months) that were better aligned with real-life scenarios. Moreover, as previously described in in vitro models, hepatotoxic effects were observed (e.g., [88]); hence, reinforcing the premise that such particles can translocate to other organs.

In vivo, smaller particles may cause greater damage, as evidenced by in vitro studies. Illustrating this, the administration of fluorescent polylactic acid oligomers revealed that fragmented particles spread through the intestines after 7 days of consumption [62]. Histological analysis reveals that they induced infiltration and inflammation in the liver, small intestine, and colon, even at low doses, which was reinforced by an increase in the pro-inflammatory cytokine tumour necrosis factor alpha. From a mechanical standpoint, inflammation was ascribed to the inactivation of matrix metallopeptidase 12 [62], an enzyme responsible for controlling the stiffness and permeability of tissues [113].

Moreover, there were concerns about potential health risks, given that they crossed the blood–brain barrier via microvascular endothelial cells [62]. It was reported that particles of both polylactic acid polymer and oligomer could remain in the digestive system for 28 days. Briefly, degradation of the polymer enhanced its bioavailability, thereby increasing global toxicity [107,108]. Polymer treatment (2.5 and 25 mg kg^−1^) led to an increase in inflammatory cell infiltration and overexpression of tumour necrosis factor alpha and interleukin-6 genes, besides increasing alanine transaminase and aspartate aminotransferase markers. Even at lower levels, polylactic acid polymer induced fibrotic damage in the liver tissue [107]. Moreover, at 6 weeks of exposure to 0.2 mg in 100 µL^−1^, these markers were enhanced, whereas micro- and nanoplastics of polylactic acid reduced antioxidant activity. Hepatocellular oedema, focal necrosis, cell vacuolisation, and karyopyknosis were noted in the liver, though the colon appeared unimpaired [109].

The oligomers were more likely to accumulate transiently; however, they degraded completely, mitigating toxicity, contrary to the previously held idea. Lower molecular weight was associated with faster in vivo degradation [108]. A 7-day exposure (50 and 500 mg kg body weight^−1^) to polyglycolic acid and poly(butylene-succinate-co-glycolate) showed that higher degradation rates were associated with lower toxicity. To illustrate, in vitro polyglycolic acid degraded more rapidly and exhibited lower toxicity. However, compared with a conventional plastic (polyethylene terephthalate), both micro- and nanoplastics derived from bioplastics had a lower impact. Exposure increased alanine transaminase levels at lower doses, whereas higher concentrations resulted in weight loss and hepatic damage [74]. Another study revealed that polylactic acid micro- and nanoplastics released from containers caused structural damage to the liver and intestines, leading to inflammatory cell infiltration and mitochondrial damage, with more pronounced effects than conventional particles over 4 weeks (0.4 and 40 mg kg^−1^) [110].

Chronic administration (3 months) of starch-based microplastics containing 40% polylactic acid (50 and 250 mg kg^−1^) resulted in hepatic oxidative stress, cellular inflammation, necrosis, and vacuolar degeneration at high doses. Exposure increased inflammatory cells and colonic necrosis, while decreasing antioxidant enzyme activity. Extended exposure may also affect colon function, as mucus production decreased over time. Prolonged exposure to environmentally relevant doses suggests that the safety of these bioplastics warrants follow-up studies before their large-scale implementation. Accordingly, research into potential health risks is urged to develop solutions to counteract these side effects. [64]. Polylactic acid polymer and oligomer treatments indirectly caused liver damage by disrupting the gut microbiota, leading to increased uric acid levels and, subsequently, excessive triglyceride accumulation. However, when particles were exposed in HepG2 cells, an increase in uric acid levels was not observed. Instead, treatment with uric acid increased *hydroxysteroid (17-beta) dehydrogenase 13* gene expression and triglyceride droplet accumulation. Moreover, after transfecting the cells with small interfering RNA, triglycerides and lipid droplets were not enhanced. These findings suggested an extra-hepatic source of uric acid. Therefore, this causality was tested in mice, whose metabolomic analysis identified a positive correlation specifically between *Bacillales* and uric acid levels. In this vein, when mice were administered with antibiotics, removal of the microbiota reduced liver damage and uric acid levels, suggesting that the microbiota is the source of uric acid production and leads to subsequent steatosis [107]. Hence, reducing toxic side effects can be attained by assuring complete degradation of micro- and nanoplastics in the digestive tract [74,107,108].

At 6-week treatment, the “central carbon metabolism in cancer” pathway was upregulated along with increased *Lachnospiraceae NK4A136*, *Lachnospiraceae A2*, and *Helicobacter* for nanoplastics, and *Lachnospiraceae NK4A136*, *Roseburia*, and *Helicobacter* for microplastics. In this way, *Lachnospiraceae NK4A136* was proposed as a potential biomarker for detecting oral exposure to micro- and nanoplastics of polylactic acid. The axis gut-microbiota-liver seems to be involved in hepatotoxicity observed in mice [109]. In the chronic exposure study, there was an increase in *Actinobacteria* (phylum), *Actinomycetia* (class), *Bifidobacteriales* (order), *Bifidobacteriaceae* (family), and *Bifidobacterium*, *Kineothrix*, and *Limosilactobacillus* (genus), while *Allobaculum* (genus) decreased. Bioplastics, therefore, require a more thorough safety assessment before their large-scale application in food packaging [64].

However, 21-day isotopic tracking following exposure to micro- and nanoplastics of polylactic acid (200 mg kg^−1^) revealed that the particles were incorporated into the carbon cycle and degraded by the microbiome, limiting translocation to distal tissues. Particles were specifically cleaved by FrsA esterase, secreted by *Helicobacter muridarum* and *Barnesiella visceriola*, whose levels were increased. The primary energy source of epithelial cells (linear fatty acids) was reduced, while L-lactic acid was converted to D-lactic acid [111], known to be linked to diabetes [114] and cognitive impairment [115], among other conditions. Although the mechanism by which microorganisms distinguish micro- and nanoplastics of polylactic acid remains unknown, a working hypothesis suggests that the microbiota may be programmed to recognise and break down these plastics [111].

Glycerophospholipid metabolism was impaired after 4 weeks of exposure, leading to disordered synthesis and breakdown of long-chain fatty acids, more pronounced than in conventional particles. The abundance of *Alistipes*, *Rikenella*, and *Turicibacter*, involved in inflammatory responses and gut barrier integrity, decreased. There was an enrichment of *Eubacterium coprostanoligenes*, *Roseburia*, and *Clostridia UCG-014* (particularly at high doses), which is also suggestive of an adaptive mechanism [110]. For instance, a study showed how polylactic acid particles ingested for 7 days led to inflammation in the liver and jejunum, which was reversed after 28 days. Evidence of dysbiosis dysfunction of the immune system was spotted after 7 days, based on the abundance of *Staphylococcus* and *Streptococcus*. However, assessment of microbiota at 28 days of exposure revealed an increase in *Bacillus*, which can protect the organism. So, it seems once again that the mice microbiota is modified in response to chronic exposure to micro- and nanoplastics derived from bioplastics [65].

## 6. Limitations

A limited number of published studies have addressed the biodegradability of bioplastics or their associated toxic effects, contributing to inconsistencies and difficulties in comparison. Crosscutting constraints across the three topics include the large heterogeneity among the types of polymers tested, whether used in their original state or degraded, different-sized particles, and the lack of uniform guidelines for characterising and analysing outcomes. Further lack of uniformity is evident in the use of different concentrations and their respective conditions (whether expressed by mass, volume, or particle count), as well as in the use of non-environmentally relevant doses. Given this inconsistency in dose parameters, applying toxicological data for risk assessment remains challenging [116].

Furthermore, the simulated in vitro digestion models varied in composition or exposure times; moreover, not all studies accounted for the entire digestion process (oral, gastric, intestinal, and colonic stages). Cell-based studies mainly focused on acute toxic effects up to 72 h, without considering chronic effects. The lack of quantification of the concentration actually reaching the cells, as well as the internalised quantity, stood out as a clear limitation. As such, although similar doses were used, interactions and responses may differ between cells, leading to a lack of comparability. Within this scope, the transport and condition of the particles upon reaching the liver received little attention in the evaluation. In addition, we should highlight the plurality of techniques used for measurement, for instance, cellular viability or oxidative stress, which requires a universal approach to study the impacts of these particles. The majority of findings were consistent with in vivo hepatotoxicity; however, the dose relevance remains uncertain as it is often extrapolated from estimates of conventional plastic exposure. In addition, chronic exposure was evaluated in only one study. Note also that the absence of particle characterisation during gastrointestinal transit was consistently observed.

Altogether, such limitations highlight the lack of guidelines and standard methodologies. So, efforts must be directed to improve their translational relevance henceforth.

## 7. Conclusions

Bioplastics emerged in response to plastic pollution as an eco-friendlier alternative [6,7,8,9,10,11]. Like conventional plastics, they can also release smaller particles [33]. Accurate information on the concentrations of ingested micro- and nanoplastics is not available, and no distinction is currently made between bioplastics and standard plastics [35]. According to estimates, up to 52,000 particles may be ingested per year, based on concentrations of micro- and nanoplastics within food (such as alcohol, bottled water, honey, seafood, salt, sugar, and tap water), and on 15% of Americans’ caloric intake, for different sexes and genders [56]. The ingestion of micro- and nanoplastics may increase the likelihood of gastrointestinal diseases, amongst others [68].

However, there are some gaps in our knowledge of the fate of bioplastics in the human digestive system. These gaps include the biotransformation of bioplastics during digestion and their (cyto)toxicity in the gastrointestinal system. To the best of our knowledge, the present review highlighted that bioplastics may not always be safer, suggesting that, in the long term, their side effects may counterbalance their benefits [41]. Overall, most studies have focused on the impact of polylactic acid on human health, primarily due to its emerging role in the food packaging market. Although other biopolymers have been tested, such studies are scarce, particularly in rodent models. Beyond that, their structures differ, with no harmonisation of the doses used, nor standardisation between methods and outcomes evaluated.

To sum up, bioplastics underwent physicochemical modifications in response to gastrointestinal fluids in in vitro models. Fragmentation can occur, resulting in pronounced porosity and cavities that facilitate new molecular interactions. For example, they could compete for lipase, thereby impairing lipid digestion over time and resulting in low levels of free fatty acids. Organic matter and biofilm-coated particles form a protein corona that also enables new molecular interactions. Microorganisms were altered, specifically *Selenomonadaceae*, *Bifidobacterium*, and *Prevotellaceae*. Moreover, marine-aged particles bioavailability for metals suggests potentially adverse impacts on human health.

Cytotoxicity was not consistently reported across all in vitro studies; however, smaller particles, those exposed to degradation conditions, and leachates appeared to have more pronounced effects. Barrier disruption was generally unimpaired despite internalisation, potentially due to lipophilic interactions. Associated outcomes included inflammation mediated by tumour necrosis factor alpha and interleukin-6 cytokines secretion, and reactive oxygen species-mediated oxidative stress, with increased alanine transaminase and aspartate aminotransferase, and impaired antioxidant enzymes.

Furthermore, hepatotoxicity was induced in vivo, particularly with smaller particles. These effects were attributed to inflammation, elevated levels of tumour necrosis factor alpha, interleukin-6, alanine transaminase, and aspartate aminotransferase, compromised hepatic function, and oxidative stress in the liver. Incomplete degradation was more harmful, influenced by higher molecular weight. A plausible indirect mechanism was proposed: an impaired microbiota, characterised by an increase in *Bacillales*, led to higher uric acid levels, which in turn caused triglyceride accumulation in the liver. Bioplastics may also be integrated into the carbon cycle, where they are broken down by the FrsA enzyme, converting lactic acid into a harmful molecule. Mechanisms of adaptation were observed following continuous exposure to bioplastics, associated with changes in the microbiota.

To conclude, it is important to note that both in vitro and in vivo models point to the potential for adverse health effects associated with bioplastic ingestion. Our review broadly outlines biotransformation in simulated models, toxic effects in relevant cells upon ingestion, and findings from rodent models. Such evidence prompts questions about the safety of commercially available bioplastics. The literature remains scarce on the toxicity of chronic and long-term exposure to bioplastics, with most studies focusing on acute exposure. However, the modulation of pathways and the observed toxicity remain largely misunderstood. While several studies demonstrate toxicity in in vitro and in vivo models, the concentrations used, particularly in in vitro systems, do not reflect real-world scenarios as they are generally higher than those estimated for human consumption. However, some in vivo studies have used doses that can be considered relevant when extrapolated from estimates of conventional plastic exposure levels. Nevertheless, it remains difficult to extrapolate the results to human health risk, especially given insufficient data on bioplastic exposure levels. For example, although new reports have documented the detection of micro- and nanoplastics, such as polylactic acid, in food and beverages, there is no clarity on the exposure concentration range or how it compares with experimental conditions. In addition, several factors can influence the comparability of these studies, and dose–response relationships must be interpreted carefully when extrapolating to human exposure.

Lastly, we emphasise the need to develop pipelines for universalising research methodologies. Hence, we propose assessing bioaccessibility, mimicking degradation and digestibility conditions before cell exposure, and employing more complex models ahead of moving to in vivo models as three-dimensional ones, in environmentally relevant conditions for longer periods, to achieve a more comprehensive assessment.

## Figures and Tables

**Table 1:** Cytotoxic effects and barrier impact of bioplastics particles and films in relevant gastrointestinal cells. The table presents studies with relevant data aligned with the objectives of our review.

Bioplastic	Exposure Conditions	Diameter Size (Methodology)	Cellular Model	Exposure Time	Exposure Dose	Cytotoxic Effects and Barrier Impacts	Reference
PLA/PBAT films	Photoaged GI conditions	853 nm in water 608 nm in GI conditions < 300 nm when collected by ultrafiltration (Zetasizer)	THP-1	24 h	0, 0.1, 1, 10, 100, 200, 500, and 1000 mg L^−1^	↓ Cell viability in a dose-dependent way EC_50_ = 495 mg L^−1^ in centrifugation collection EC_50_ = 243 mg L^−1^ in ultrafiltration collection ↑ Cytotoxic effects in ultrafiltration-derived MNPs, leading to death at the highest concentrations Cytotoxicity was similar to or higher than that of conventional plastics	[69]
PLA-MNPs	Pristine	2733 nm and 300 nm ^†^ (Zetasizer)	HepG2, HepaRG, and Caco-2 Transwell	2, 4, 6, 24, 48, and 72 h	5 × 10^7^ to 2.5 × 10^10^ µm^2^ particles mL^−1^	↑ Cytotoxic effects of smaller particles at high concentrations in HepG2 cells at 24 h ↑ Toxic responses observed in smaller particles after 24 h in Caco-2, HepaRG and HepG2 cells Permeability barrier was not affected ↑ Uptake of smaller particles	[88]
PLA-NPs	Simulation of tea preparation (mechanical and thermodynamic stresses)	Non-sonicated 395.09 ± 381.08 nm in water ^†^ Sonicated 266.74 ± 132.46 nm in water 281.50 ± 138.65 nm in DMEM medium (DLS) Non-sonicated 159.48 ± 6.06 nm in water ^†^ Sonicated 113.65 ± 8.43 nm in water 116.50 ± 7.00 nm in DMEM medium (TEM)	Undifferentiated Caco-2 and HT29 monoculture Differentiated Caco-2/HT29 barrier co-culture	3, 24, 48, and 72 h	0, 50, and 100 µg mL^−1^	Cell viability was not affected in monoculture models at 100 µg mL^−1^ up to 48 h ROS levels induction was not observed HT29 cells internalised all PLA-NPs (up to 72h) Caco-2 cells internalised 60% at 48 h ↓ TEER observed after 3 h	[83]
PLA-MPs	Thermal simulation	250.99 nm (DLS)	Caco-2	48 h	NR	↓ Cell viability after exposure to raw leachate and supernatant ROS levels induction was not observed	[89]
PBAT-modified starch blended film	Food simulants	NR	L-02	48 h	0, 0.025, 0.05, 0.1, 0.2, 0.3, 0.4, and 0.5 mg mL^−1^ of extract migration	↓ Cell viability at 10% and 20% (*v*/*v*) ethanol starting at 0.4 mg mL^−1^ ↓ Viability at 4% (*v*/*v*) acetic acid, and at 50% and 95% (*v*/*v*) ethanol starting at 0.3, 0.2, and 0.05 mg mL^−1^, respectively ↑ Biomarkers of liver damage ↑ Pro-inflammatory cytokine ↓ Antioxidant enzymes ↑ ROS levels	[84]
PLA-MNPs	GI conditions	2733 nm and 300 nm ^†^ (evaluated before) [88] 27 and 30 µm (aggregates) in cell culture medium (Zetasizer)	Caco-2 Transwell	24 h	3.00 × 10^10^ and 2.40 × 10^11^ µm^2^ particles mL^−1^, and diluted 1:40, 1:20, and 1:20	Cell viability was not affected ↑ Cellular interaction of undigested particles Barrier integrity was not affected ↑ Uptake of smaller digestate particles compared with undigested ones	[90]
PLA- and PHB-MNPs	Additives extraction	100 nm to 10 µm ^†^ (SEM)	HepG2	24 h	0.781 to 50% and 0.55 to 100% of extracts for cell viability and ROS assays, respectively	↓ Cell viability in a dose-dependent way up to 15% (not statistically significant) ↑ Oxidative stress in a dose-dependent way in some cases	[82]
PGA, aged PGA, PBS, PBC, PBAT and PLA particles	PGA naturally aged Soil migration simulation	700–800 nm in water (DLS)	LO2, Caco-2, and THP-1	24, 48, and 72 h	1, 25, and 100 mg L^−1^	PGA, PBS, PBAT, PLA, and PBC had antiproliferative effects in LO2 cells PGA and aged PGA showed a lower reduction in viability Antiproliferative effects were not observed in Caco-2 cells Antiproliferative effects in THP-1 cells were observed THP-1 cells internalised PGA particles by endocytosis	[67]
PBAT films	Combination of additives Photoaged Soil exposure	NR	HepG2/C3A	24 h	100, 250, 500, 750, and 1000 µL mL^−1^	↓ Cell viability in a dose-dependent way in all conditions Up to 250 µL mL^−1^ remained above 80% Genotoxicity and micronuclei were not induced at 250 µL mL^−1^	[91]
PLA-MNPs	Pristine	2733 nm and 300 nm ^†^ (evaluated before) [88] (Zetasizer)	Caco-2, HepG2 Caco-2/Raji-B, Caco-2/HT29-MTX, Caco-2/HepaRG	24 h	1 × 10^8^, 5 × 10^8^, and 2.5 × 10^9^ µm^2^ particles mL^−1^	Cell viability was evaluated before (without cytotoxicity) [88] ↑ Uptake of smaller particles Integrity and permeability were not compromised ↑ Pro-inflammatory cytokines in Caco-2 and HepaRG cells	[87]
PLA-MNPs	Pristine	317 ± 27 in water nm ^†^ 259 ± 14 nm in intestinal cell differentiation medium ^†^ (DLS)	iPSCs differentiated into intestinal epithelial cell layers	24 h	125 µL mL^−1^	Cell viability was not affected ROS levels induction was not observed No differences found in the secretion of pro-inflammatory cytokines Epithelium internalised PLA Barrier integrity was not affected	[92]
PLA- and CA-MNPs	Pristine	<3 µm in 1 mM KCl solution and DMEM medium ^†^ (DLS)	lmKC, J774A.1, STC-1, and BNL CL.2	24 h	1–100 particles cell^−1^	Cytotoxic effects were not observed ↓ Metabolic activity in BNL CL.2 cells after exposure to PLA ↑ ROS levels in a dose-dependent way after exposure to PLA in J744A.1 and lmKC cells ↑ ROS levels after exposure to CA in all cells J774A.1 and lmKC cells ingested PLA and CA BNL CL.2 cells ingested CA	[77]

PLA, polylactic acid; PBAT, polybutylene adipate terephthalate; GI, gastrointestinal; EC_50_, half maximal effective concentration; MNPs, micro- and nanoplastics; NPs, nanoplastics; DMEM, Dulbecco’s Modified Eagle Medium; DLS, dynamic light scattering; TEM, transmission electron microscopy; ROS, reactive oxygen species; TEER, transepithelial electrical resistance; MPs, microplastics; NR, not reported; PHB, polyhydroxybutyrate; SEM, scanning electron microscope; PGA, polyglycolic acid; PBS, polybutylene succinate; PBC, poly(butylene carbonate); iPSCs, induced pluripotent stem cells; CA, cellulose acetate. ^†^ Pristine characterisation; ↑ increased; ↓ decreased.

**Table 2:** Toxic effects of ingested bioplastic particles in murine models on the digestive system. The table shows selected studies containing key variables consistent with the aims of our review.

Bioplastic	Diameter Size (Methodology)	In Vivo Model	Exposure Time	Exposure Dose	Toxic Effects	Reference
PLA polymer and oligomer	25.1 µm ^†^ (SEM)	Mice	7 days	0.01, 0.1 and 1.0 mg day^−1^ diary oral gavage	↑ Inflammation and infiltration in liver, small intestine, and colon ↑ TNF-α in liver, small intestinal, and colon ↓ Mucus in the small intestine and colon at a lower dose	[62]
PLA polymer and oligomer	NR	C57BL/6J mice	28 days	2.5 and 25 mg kg^−1^ oligomer and polymer diary oral gavage	↑ Hepatic inflammation ↑ Inflammation and damage caused by polymer particles ↑ Uric acid in liver ↑ Triglycerides and lipid droplets Alteration in gut microbiota	[107]
PLA polymer and oligomer	2.5 µm ^†^ Faeces: 200, 100, and <100 nm for 1, 14, and 28 days, respectively (DLS)	C57BL/6J mice	1, 14, and 28 days	2.5 and 25 mg kg^−1^ diary oral gavage	↑ Biodistribution of PLA polymer ↑ Accumulation of PLA oligomer ↑ Toxicity is associated with incomplete degradation of polymer Specific toxicity in the digestive system was not assessed	[108]
PLA-MPs and PLA-NPs	50 nm and 5 µm ^†^ (NR)	Institute of Cancer Research mice	6 weeks	0.2 mg 100 µL^−1^ diary oral gavage	↑ Liver damage biomarkers ↓ Total antioxidant capacity Hepatotoxicity Dysbiosis intestinal Metabolic alterations in gut	[109]
PGA- and PBSG-MPs	50 µm ^†^ (SEM)	Wistar rat	7 days	50 and 500 mg kg body weight^−1^ diary oral gavage	↓ Liver and stomach weight ↑ Liver damage biomarkers ↑ Pathological alterations at higher doses Inflammation in liver at higher doses	[74]
PLA-MPs	1 to 30 µm ^†^ (stereomicroscopy)	C57BL/6J mice	4 weeks	0.4 and 40 mg kg^−1^ in diet	↑ Impact on metabolic pathways at high dose group Glycerophospholipid metabolism impaired ↑ Liver cell nuclear aggregation, mitochondrial damage, and inflammatory cell infiltration at high dose group ↑ Damage in gut and liver than conventional plastics Alterations in the abundance of intestinal microbiota	[110]
Starch-based MPs	4–800 µm ^†^ (particle size analyser)	Mice	3 months	50 and 250 mg kg^−1^ diary food	↑ Hepatic oxidative ↑ Lipid metabolism dysfunction Cellular inflammation, necrosis, and vacuolar degeneration in liver ↑ Lipids in liver ↑ Oxidative stress in liver ↑ Infiltration of inflammatory cells and necrosis in colon ↓ Mucus in colon Alterations in composition of microbiota	[64]
PLA-MPs	50.43 ± 24.12 µm ^†^ (SEM)	C57BL/6 mice	21 days	200 mg kg^−1^ oral gavage	PLA fragments entered the tricarboxylic acid cycle in intestinal epithelial cells ↓ Short acid fatty Impaired gut metabolism Impaired gut barrier	[111]
PLA-MPs	2–5 µm ^†^ Faeces: 600–700 and 300–400 nm for 7 and 28 days, respectively (TEM)	BALB/c mice	7 and 28 days	50 mg kg body weight^−1^ day^−1^ diary gavage	↑ Inflammatory responses in gut and liver dependent on time ↓ Alpha microbiota	[65]

PLA, polylactic acid; SEM, scanning electron microscope; TNF-α, tumour necrosis factor-alpha; NR, not reported; DLS, dynamic light scattering; MPs, microplastics; NPs, nanoplastics; PGA, polyglycolic acid; PBSG, poly(butylene succinate-co-glycolate); TEM, transmission electron microscopy. ^†^ Pristine characterisation; ↑ increased; ↓ decreased.

## Data Availability

No new data were created or analyzed in this study. Data sharing is not applicable to this article.
